# Highly Uncontrolled Cardiovascular Risk in Emerging Adults With Paediatric‐Onset Type 1 Diabetes—A Cross‐Sectional Analysis From the Diabetes Prospective Follow‐Up Registry DPV

**DOI:** 10.1111/dom.70610

**Published:** 2026-03-16

**Authors:** Alena Welters, Christina Reinauer, Karl Otfried Schwab, Claudia Boettcher, Melanie Hess, Heike Bartelt, Axel Dost, Joachim Rosenbauer, Angela Galler, Reinhard W. Holl

**Affiliations:** ^1^ Department of General Pediatrics, Neonatology and Pediatric Cardiology, Medical Faculty and University Hospital Düsseldorf Heinrich Heine University Düsseldorf Düsseldorf Germany; ^2^ Medizinisches Versorgungszentrum (MVZ) Clotten, Limbach‐Group Germany; ^3^ Paediatric Endocrinology and Diabetology, University Children's Hospital, Julie‐von‐Jenner Haus University of Bern Bern Switzerland; ^4^ Department of Pediatric Endocrinology and Diabetology, University Children's Hospital University of Basel Basel Switzerland; ^5^ Hospital for Children and Adolescents University of Leipzig Leipzig Germany; ^6^ Department of Paediatrics and Adolescent Medicine University Hospital Jena Jena Germany; ^7^ Institute for Biometrics and Epidemiology, German Diabetes Center (DDZ) Leibniz Center for Diabetes Research at Heinrich Heine University Düsseldorf Düsseldorf Germany; ^8^ German Center for Diabetes Research (DZD) München‐Neuherberg Germany; ^9^ Charité—Universitätsmedizin Berlin, Corporate Member of Freie Universität Berlin and Humboldt‐Universität Zu Berlin Sozialpädiatrisches Zentrum, Paediatric Endocrinology and Diabetology Berlin Germany; ^10^ Institute of Epidemiology and Medical Biometry, ZIBMT University of Ulm Ulm Germany

**Keywords:** cardiovascular disease, diabetes complications, dyslipidaemia, glycaemic control, type 1 diabetes

## Abstract

**Aims:**

Cardiovascular disease (CVD) is a major contributor to premature morbidity and mortality in individuals with type 1 diabetes (T1DM). Glycaemic control often deteriorates during the transition from paediatric to adult care, and thus we assessed the prevalence and pharmacological management of overt and actionable modifiable cardiovascular risk factors in adolescents and young adults with paediatric‐onset T1DM. We examined factors associated with risk burden and early microvascular complications.

**Methods:**

We analysed data from 7298 individuals aged 17–26 years with paediatric‐onset T1DM and ≥ 2 years diabetes duration in the DPV registry between 2020 and 2023. Five predefined CVD risk factors were assessed (HbA1c > 9%, obesity, elevated blood pressure, LDL > 130 mg/dL, smoking), using median values over a 3‐year observation period. Factors associated with cumulative risk burden and associations with retinopathy and microalbuminuria were evaluated using multivariable linear and logistic regression models.

**Results:**

At least one CVD risk factor was present in 49.2% of individuals; 19.1% had ≥ 2 and 5.2% ≥ 3 risk factors. Poor glycaemic control, defined as HbA1c > 9% (21.8%) and elevated blood pressure (17.6%) was most frequent. CVD risk factor burden was associated with diabetes duration > 10 years and migration background. The likelihood of microalbuminuria increased progressively with the number of CVD risk factors, with an odds ratio of 2.68 (95% CI: 1.34–5.36) among individuals with four risk factors. Within this high‐risk cohort, significant indication‐treatment gaps remained: only 9.9% with overtly elevated LDL cholesterol > 130 mg/dL and 19.8% with hypertension > 140/90 mmHg received medication.

**Conclusions:**

Young adults with paediatric‐onset T1DM show a high burden of modifiable CVD risk factors with substantial treatment gaps, and clustering is associated with microvascular complications. Early identification and targeted intervention are critical to mitigating long‐term vascular damage.

## 1 Introduction

Type 1 diabetes mellitus (T1DM) is one of the most common chronic metabolic disorders in childhood and adolescence, affecting approximately 8.5 million individuals worldwide, including 1.5 million people under the age of 20 [1, 2]. Although advancements in diabetes care have significantly improved life expectancy, individuals with T1DM still face an increased risk of premature morbidity and mortality, predominantly because of cardiovascular disease (CVD). Epidemiological studies have shown that people with T1DM have a 2‐ to 5‐fold increased risk of death, with CVD being a major contributing factor [3, 4, 5, 6, 7, 8, 9]. The younger the age at onset, the greater the excess mortality risk. Individuals diagnosed with T1DM before the age of 10 experience a significant reduction in life expectancy, with men losing 14.2 years and women losing 17.7 years [4]. Although even HbA1c levels below the recommended guideline threshold of 7.0% (53 mmol/mol) are associated with at least a twofold increased risk of death from any cause or from cardiovascular causes [10], recent large‐scale epidemiological studies suggest that cardiometabolic risk factors, such as poor glycaemic control, hypertension, dyslipidaemia, smoking and albuminuria are the strongest factors associated with mortality and CVD among individuals with T1DM [3, 11]. A Mendelian randomisation study found that, after adjusting for confounding factors such as hypertension and dyslipidaemia, T1DM remained significantly associated only with atherosclerosis, whereas no direct causal relationship was observed with other CVDs, including myocardial infarction, stroke and coronary artery disease [12]. In line with these findings, controlling modifiable risk factors in individuals with T1DM is strongly associated with a reduced risk of cardiovascular (CV) events and mortality [3, 11, 13].

Adolescence and early adulthood represent a critical window for the emergence and accumulation of cardiometabolic risk. During this time, diabetes care shifts from being predominantly parent‐ or caregiver‐directed to being self‐managed, requiring increased autonomy, responsibility and engagement from the affected individual [14, 15]. This developmental period, including the transition from paediatric to adult health care, is often marked by gaps in care, poor treatment adherence, and deteriorating glycaemic control, with HbA1c levels often exceeding guideline‐recommended targets [16, 17, 18, 19, 20]. At the same time, traditional CVD risk factors, such as hypertension, dyslipidaemia, obesity, smoking and alcohol use, begin to surface [21, 22]. Despite the well‐documented role of these risk factors in driving CVD‐related morbidity, studies suggest that their awareness and management in individuals with T1DM remain insufficient [23, 24, 25, 26, 27, 28]. In this study, we assessed the prevalence and management of modifiable, preventable CVD risk factors in adolescents and young adults with T1DM, using data from the Diabetes Prospective Follow‐Up (DPV) registry. This analysis aimed to identify individuals clearly exceeding clinically relevant CVD risk thresholds, regardless of whether pharmacological treatment had been initiated.

## 2 Materials and Methods

### 2.1 Data Source and Study Population

Data were obtained from the DPV registry, a multicentre initiative that prospectively collects demographic and clinical data of individuals with all types of diabetes across Germany, Austria, Switzerland and Luxembourg. Participating centres, covering all levels of care, submit anonymised patient data biannually to the University of Ulm, Germany, where data are centrally aggregated and analysed. As of March 2024, the DPV registry included 699 105 individuals with any form of diabetes mellitus. The registry provides high coverage of the target population, capturing an estimated > 90% of paediatric individuals and > 70% of young adult individuals with T1DM in Germany [29]. In total, more than 500 centres actively contribute to the registry. In the present analysis, anonymised data from 356 centres were included. The DPV initiative and the analysis of anonymised data on quality of care are approved by the ethics committee of the University of Ulm (reference no. 314/21) and by local review boards of the participating centres.

We included individuals with a clinical diagnosis of T1DM who were aged 17–26 years with diabetes onset before the age of 17 years (paediatric‐onset) and a diabetes duration of more than 2 years. The analysis included data from the treatment years 2020–2023 to reflect contemporary clinical practice and current data availability. Only individuals with documented smoking status, binary sex assignment (male/female), and available data across all five predefined CVD risk domains were included. The final study population comprised 7298 individuals (Figure S1).

### 2.2 Demographic and Clinical Variables

Demographic and clinical data were extracted from the DPV registry including age, sex, age at diabetes onset, diabetes duration, migration background, HbA1c, body mass index (BMI), systolic and diastolic blood pressure (BP), lipid profiles (total cholesterol, LDL‐, HDL‐ and non‐HDL‐cholesterol, triglycerides), frequency of microalbuminuria, presence of diabetic retinopathy, and use of antihypertensive or lipid‐lowering medications. Documented medication use reflects physician‐reported prescriptions at the most recent visit and does not include information about adherence, dispensing, or treatment duration. BMI was calculated as body weight kg divided by the square of body height m (kg/m^2^). HbA1c values were mathematically standardised to the Diabetes Control and Complications Trial (DCCT) reference range (4.05%–6.05%) using the multiple‐of‐the‐mean method to ensure comparability across centres. Laboratory parameters were considered comparable due to national quality standards (RiliBÄK, guidelines of the German Medical Association for quality assurance in laboratory medicine) and systematic participation in external proficiency testing. A positive migration background was defined as the patient or at least one parent born outside of Germany, Austria, Switzerland or Luxembourg.

The five predefined modifiable CVD risk factors were poor glycaemic control (HbA1c > 9.0%; 75 mmol/mol), obesity (BMI > 30 kg/m^2^), dyslipidaemia (median LDL cholesterol > 130 mg/dL), smoking and elevated BP. Two cut‐offs for BP are reported: elevated BP was defined as a median systolic BP ≥ 130 mmHg or diastolic BP ≥ 80 mmHg, whereas arterial hypertension warranting pharmacological treatment was defined as a median systolic BP ≥ 140 mmHg or diastolic BP ≥ 90 mmHg, with medians calculated over the 3‐year observation period. Information about e‐cigarette or vaping product use was not available. All five risk factors were evaluated after the remission phase (i.e., diabetes duration of more than 2 years). Retinopathy was documented as the physician‐reported presence of non‐proliferative or proliferative diabetic retinopathy. Microalbuminuria was defined as a median urinary albumin‐to‐creatinine ratio > 30 mg/g creatinine across all available measurements, as a pragmatic approximation of guideline‐based definitions of persistent albuminuria. In addition to assessing the prevalence of CVD risk factors, we evaluated the use of pharmacological therapies in individuals meeting the criteria for dyslipidaemia and arterial hypertension. Specifically, we assessed the proportion of individuals with dyslipidaemia (LDL cholesterol > 130 mg/dL) or hypertension (systolic BP ≥ 140 mmHg or diastolic BP ≥ 90 mmHg) who received lipid‐lowering or antihypertensive medication, respectively, based on established guidelines. Each subgroup (treated vs. untreated individuals) was further stratified by demographic and clinical variables, including the number of CVD risk factors, to explore potential differences. We also examined the use of metformin in individuals with obesity (BMI > 30 kg/m^2^).

### 2.3 Statistical Analysis

All analyses were performed using SAS version 9.4 (build Ts1M6, SAS Institute Inc., Cary, NC, USA) on a Windows server mainframe. Descriptive statistics are presented as medians with interquartile ranges (IQR) for continuous variables and as absolute and relative frequencies for categorical variables. Group comparisons were performed using the Chi‐square test for categorical variables and the Wilcoxon test for continuous variables, as appropriate. A two‐sided *p*‐value of less than 0.05 was considered statistically significant.

To identify factors associated with the number of CVD risk factors (range: 0–5), we conducted multivariable linear regression analyses. Independent variables included sex, migration background, age at diabetes onset and diabetes duration, including an interaction term between age at onset and diabetes duration. For microalbuminuria, CSII use was additionally included as a covariate. To minimise collinearity, diabetes duration (categorised as 2–5, 5–10 and > 10 years) and age at diabetes onset (categorised as prepubertal vs. [post‐]pubertal) were modelled as categorical variables rather than continuous measures, and current age was not included in multivariable models. Due to limited documentation of pubertal onset in the DPV registry, classification was based on age at diabetes onset: for girls, prepubertal < 11 years, pubertal 11–16 years and postpubertal > 16 years; for boys, prepubertal < 12 years, pubertal 12–17 years and postpubertal > 17 years. Associations between the number of CVD risk factors and microvascular complications (retinopathy and microalbuminuria) were assessed using multivariable logistic regression models, reporting odds ratios (OR) with 95% confidence intervals and *p*‐values.

## 3 Results

### 3.1 Clinical Characteristics and Prevalence of Cardiovascular Risk Factors Among Young Adults With T1DM

The study population included 7298 young adults (46.2% female) with paediatric‐onset T1DM, aged 17–26 years (median age: 17.8 years, IQR: 17.5–18.5), and a median diabetes duration of 8.5 years (IQR: 5.5–12.2). A migration background was documented in 23.5% of individuals. Table 1 summarises the clinical characteristics and the prevalence of the five predefined modifiable CV risk factors. Of individuals with complete documentation of all 5 risk domains, 21.8% had poor glycaemic control (HbA1c > 9.0%), 17.6% had elevated blood pressure (> 130/90 mmHg), 14.0% had elevated LDL cholesterol levels (> 130 mg/dL), 13.3% were current smokers, and 7.5% were classified as obese (BMI > 30 kg/m^2^). Nearly half of all individuals (49.2%) had at least one CVD risk factor. A total of 19.1% had two or more risk factors, whereas 5.2% and 0.9% had ≥ 3 and ≥ 4 CVD risk factors, respectively. Among the 7298 individuals with all five risk factors documented, 103 (1.4%) received metformin, 393 (5.4%) received any antihypertensive medication—predominantly an ACE inhibitor (*n* = 277; 3.8%)—and 188 individuals (2.6%) received lipid‐lowering therapy, almost exclusively statins (*n* = 181; 2.5%), occasionally in combination with ezetimibe. So, while many achieved recommended therapeutic targets, the present study specifically addressed treatment gaps and undertreatment.

**TABLE 1:** Clinical characteristics of individuals with T1DM with documentation of all five risk domains.

	*n*	Median (Q1–Q3) or %
Age (years)	7298	17.8 (17.5–18.5)
Male	7298	53.9
Age at diabetes onset (years)	7298	9.6 (6.0–12.6)
Diabetes duration (years)	7298	8.5 (5.5–12.2)
Migrational background	7298	23.5
Appointments per individual	7298	5 (4–8)
BMI (kg/m^2^)	7298	23.7 (21.5–26.5)
Overweight (BMI > 25 kg/m^2^)	7298	25.3
**Obesity (BMI > 30 kg/m^2^)**	7298	**7**.**5**
Systolic blood pressure (mmHg)	7298	126 (120–132)
Diastolic blood pressure (mmHg)	7298	75 (70–80)
**Elevated blood pressure (> 130/80 mmHg)**	7298	**17**.**6**
Arterial hypertension (> 140/90 mmHg)	7298	1.8
Total cholesterol (mg/dL)	7224	168 (147–191)
LDL cholesterol (mg/dL)	7298	96 (78–116)
HDL cholesterol (mg/dL)	7214	57 (49–67)
Non‐HDL cholesterol (mg/dlL)	7151	109 (89–131)
Triglycerides (mg/dL)	7110	92 (66–134)
**LDL cholesterol > 130 mg/dL**	7298	**14.1**
HbA1c (%)	7298	7.9 (7.2–8.9)
HbA1c > 7%	7298	79.8
**HbA1c > 9% (> 75 mmol/mol) (%)**	7298	**21.8**
**Smoker**	7298	**13.3**
Diabetic retinopathy	4306	0.3
Microalbuminuria	6012	10.0
**% ≥ 1 CVD risk factors**	7298	**49.2**
**% ≥ 2 CVD risk factors**	7298	**19.1**
**% ≥ 3 CVD risk factors**	7298	**5.2**
**% ≥ 4 CVD risk factors**	7298	**0**.**9**

*Note*: Demographic, clinical characteristics and prevalence of predefined modifiable cardiovascular risk factors in the entire cohort (*n* = 7298). Continuous variables are reported as median (interquartile range). Percentages indicate the proportion of individuals with values above predefined thresholds or with specific categorical characteristics (e.g., migration background, smoking status). The number of individuals (*n*) refers to the number for whom data were available for the respective variable. Cardiovascular risk factors include poor glycaemic control (HbA1c > 9%), elevated blood pressure (systolic > 130 mmHg and/or diastolic > 80 mmHg), elevated LDL cholesterol (> 130 mg/dL), obesity (BMI > 30 kg/m^2^) and active smoking.

### 3.2 Subgroup Differences in CVD Risk Factor Prevalence Among Young Adults With T1DM

**FIGURE 1:**
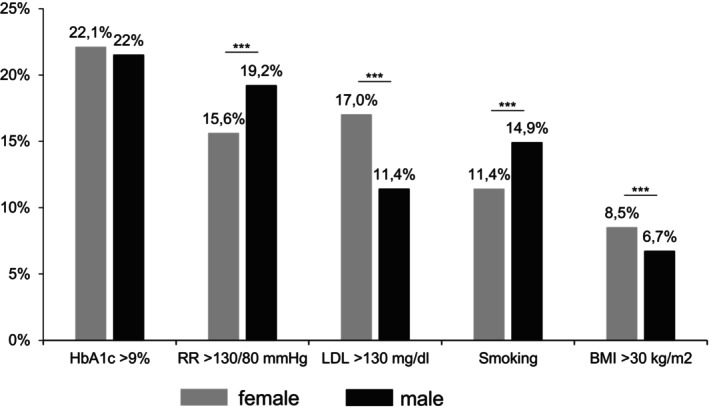
Sex‐specific prevalence of predefined cardiovascular risk factors in young adults with type 1 diabetes. Prevalence of five predefined modifiable cardiovascular risk factors (poor glycaemic control, elevated blood pressure, elevated LDL cholesterol, obesity and smoking) stratified by sex in 7.298 individuals with paediatric‐onset type 1 diabetes aged 17–26 years. Significance determined by *p* < 0.05 using *χ* ^2^‐test. ***p < 0.001.

Figure 1 illustrates the sex‐specific prevalence of the five predefined CVD risk factors. Compared with females, males had a significantly higher prevalence of elevated blood pressure (19.2% vs. 15.6%), and were more often active smokers (14.9% vs. 11.4%). In contrast, elevated LDL cholesterol and obesity were more common among females (17.0% vs. 11.4%, and 8.5% vs. 6.7%, respectively). No significant differences between sexes were observed in the prevalence of poor glycaemic control. Further subgroup analyses are shown in Figure S2a,b. CVD risk factor prevalence was similar between individuals with and without a migration background (Figure S2a). However, poor glycaemic control (HbA1c > 9%) was more common among individuals with a migration background compared to those without (25.5% vs. 20.7%, *p* < 0.05). Similarly, when stratified by age at diabetes onset (prepubertal vs. pubertal; Figure S2b), the prevalence of poor glycaemic control was significantly higher among those with prepubertal onset (23.9% vs. 17.9%, *p* < 0.05), whereas the prevalence of other risk factors did not differ significantly between groups. Despite the use of advanced diabetes technologies, almost 20% of CSII or automated insulin delivery (AID) treated individuals continued to have HbA1c levels > 9% (Figure S2c,d). Although LDL cholesterol was significantly lower in CSII than in MDI and even lower in AID users, new technologies did not correspond to fewer risk factors, with almost 20% of patients in this age category presenting with an HbA1c > 9%.

### 3.3 Determinants of CVD Risk Factor Burden

To explore potential determinants of CVD risk accumulation, we performed multiple linear regression analyses with the total number of predefined CVD risk factors (range 0–5) as the dependent variable. Independent variables included sex, migration background, age at diabetes onset (categorised as prepubertal vs. pubertal/postpubertal onset), and diabetes duration (categorised as 2–5, 5–10 and > 10 years). A significantly greater burden of CVD risk factors was observed in patients with a diabetes duration exceeding 10 years compared with those with 2–5 years of disease duration (*p* = 0.002). No significant difference was found for individuals with a diabetes duration of 5–10 years (*p* = 0.57). In addition, having a migration background was significantly associated with a higher number of CVD risk factors (*p* = 0.031), compared to individuals without a migration background. In contrast, no significant associations were observed for sex or age at diabetes onset.

### 3.4 Pharmacological Management of CVD Risk Factors

Among individuals with elevated LDL cholesterol (median > 130 mg/dL over the 3‐year observation period; *n* = 1023), only 9.9% (*n* = 101) received lipid‐lowering therapy based on the most recently documented treatment status (Table 2a). Although no significant differences were observed in age, sex, diabetes duration, or migration background between treated and untreated individuals, those receiving treatment had higher median levels of total cholesterol (243 vs. 225 mg/dL), LDL cholesterol (163 vs. 156 mg/dL), non‐HDL cholesterol (187 vs. 167 mg/dL) and random, non‐fasting triglycerides (218 vs. 109 mg/dL; all *p* < 0.01). They also more frequently exceeded the non‐HDL cholesterol threshold of 160 mg/dL (75.8% vs. 55.0, *p* < 0.01). Use of antihypertensive medication (16.8% vs. 7.5%; *p* < 0.05) and metformin (8.9% vs. 1.5%, *p* < 0.01) was significantly more common among statin‐treated individuals compared to untreated individuals. No significant differences were observed between groups with regard to the prevalence of the other predefined CVD risk factors or the total number of risk factors present.

**TABLE 2:** (a–c) Pharmacological management and cardiovascular risk profiles in individuals with dyslipidemia (a), arterial hypertension (b) and obesity (c).

(a) Individuals with LDL‐hypercholesterolemia (> 130 mg/dL)			
Lipid‐lowering medication status	Yes (*n* = 101; 9.9%)	No (*n* = 922; 90.1%)	*p*
Age (*y*)	17.9 (17.5–19.6)	17.7 (17.5–18.4)	n.s.
Male (%)	38.6	44.5	n.s.
Diabetes duration (*y*)	9.4 (6.8–12.9)	8.8 (5.6–12.3)	n.s.
Migrational background (%)	21.8	24.1	n.s.
BMI (kg/m^2^)	26.7 (24.3–29.6)	25.3 (22.6–29)	n.s.
Total cholesterol (mg/dL)	237 (220–264)	221 (206–243)	< 0.01
LDL cholesterol (mg/dL)	157 (143–176)	146 (137–162)	< 0.01
HDL cholesterol (mg/dL)	55 (47–64)	56 (48–66)	n.s.
Non‐HDL cholesterol (mg/dL)	182 (161–211)	163 (155–182)	< 0.01
Non‐HDL cholesterol > 160 mg/dL (%)	76.4	54.6	< 0.01
Triglycerides (mg/dL)	166 (115–249)	138 (100–198)	< 0.01
Overweight (BMI > 25 kg/m^2^, %)	52.3	38.8	0.07
Obesity (BMI > 30 kg/m^2^; %)	16.9	15.2	n.s.
Elevated BP (%)	30	25.6	n.s.
HbA1c > 7% (> 53 mmol/mol; %)	92.1	90.5	n.s.
HbA1c > 9% (> 75 mmol/mol; %)	43.1	38.6	n.s.
Smoker (%)	16.9	12	n.s.
Number of risk factors	2 (1–3)	2 (1–2)	n.s.
Antihypertensive medication (%)	16.9	7.0	< 0.01
Metformin (%)	7.7	1.4	< 0.01

(b) Individuals with hypertension (BP > 140/90 mmHg)			
Antihypertensive medication	Yes (*n* = 26; 19.8%)	No (*n* = 105; 80.2%)	*p*
Age (*y*)	18.6 (17.7–20.1)	17.9 (17.6–18.6)	n.s.
Male (%)	76.9	59.1	n.s.
Diabetes duration (*y*)	8.8 (6.0–10.8)	0.7 (5.6–12.7)	n.s.
Migrational background (%)	11.5	22.9	n.s.
Systolic blood pressure (mmHg)	149 (145–152)	145 (142–150)	n.s.
Diastolic blood pressure (mmHg)	93 (91–98)	93 (90–97)	n.s.
BMI (kg/m^2^)	27.8 (24.7–333.4)	26.6 (23.4–30.4)	n.s.
Overweight (BMI > 25 kg/m^2^, %)	57.7	52.4	n.s.
Obesity (BMI > 30 kg/m^2^; %)	38.5	17.1	n.s.
LDL > 100 mg/dL (%)	57.7	57.1	n.s.
LDL > 130 mg/dL (%)	23.1	24.8	n.s.
HbA1c > 7% (> 53 mmol/mol)	84.6	81.9	n.s.
HbA1c > 9% (> 75 mmol/mol)	38.5	24.8	n.s.
Smoker (%)	19.2	12.4	n.s.
Number of risk factors	2 (2–3)	2 (1–2)	n.s.
Lipid‐lowering‐medication (%)	15.4	1.0	0.018
Metformin (%)	19.2	1.9	0.013

(c) Individuals with obesity (BMI > 30 kg/m^2^)			
Metformin	Yes (*n* = 55; 10.1%)	No (*n* = 490; 89.9%)	*p*
Age (*y*)	17.8 (17.5–18.9)	17.8 (17.5–18.6)	n.s.
Male (%)	52.7	46.9	n.s.
Diabetes duration (*y*)	8.1 (4.5–11.9)	8.3 (5.6–11.7)	n.s.
Migrational background (%)	29.1	27.4	n.s.
BMI (kg/m^2^)	35.6 (33.8–39.2)	33.5 (32.2–36.1)	< 0.01
LDL > 100 mg/dL (%)	67.3	68.0	n.s.
LDL > 130 mg/dL (%)	27.3	28.6	n.s.
Elevated BP > 130/80 mmHg (%)	36.4	33.3	n.s.
HbA1c > 7% (> 53 mmol/mol; %)	84.6	81.9	n.s.
HbA1c > 9% (> 75 mmol/mol; %)	38.2	24.1	n.s.
Smoker (%)	12.1	13	n.s.
Number of risk factors	2 (1–3)	2 (1–3)	n.s.
Lipid‐lowering‐medication (%)	10.9	4.9	n.s.
Antihypertensive medication (%)	27.3	13.3	n.s.

*Note*: Clinical characteristics and cardiovascular risk profiles between treated and untreated individuals with (a) dyslipidemia (LDL cholesterol > 130 mg/dl), (b) arterial hypertension (blood pressure > 140/90 mmHg) and (c) obesity (BMI > 30 kg/m^2^). Data are presented as median (interquartile range) for continuous variables and as percentages for categorical variables. Significance determined by *p* < 0.05 using Wilcoxon test for continuous variables or *χ* ^2^‐test for categorial variables.

Abbreviation: n.s., not significant.

Among individuals with arterial hypertension (> 140/90 mmHg; *n* = 131), no significant differences in age, sex, diabetes duration or migration background were observed between those with and without antihypertensive medication. In this subgroup, only 19.8% (*n* = 26) received antihypertensive therapy (Table 2b). Compared to untreated individuals, those receiving treatment more frequently also received metformin (19.2% vs. 1.9%) and lipid‐lowering therapy (15.4% vs. 1.0%, *p* < 0.05). No significant differences were found between groups regarding the prevalence of the other predefined CVD risk factors or in the total number of risk factors present.

Among individuals with obesity (BMI > 30 kg/m^2^; *n* = 545), metformin use was documented in 10.1% (*n* = 55; Table 2c). Treated individuals had a significantly higher median BMI (36.7 vs. 34.6 kg/m^2^, *p* < 0.01). Numerically higher rates of poor glycaemic control (HbA1c > 9%: 38.2% vs. 24.1%), as well as more frequent use of antihypertensive (27.3% vs. 13.3%) and lipid‐lowering therapy (10.9% vs. 4.9%) were observed in obese patients; however, none of the observed differences reached statistical significance.

### 3.5 Association Between the Number of CVD Risk Factors and Microvascular Complications

To investigate the relationship between cumulative CVD risk burden and the presence of microvascular complications, we performed logistic regression analyses using the number of predefined CVD risk factors as the independent variable.

In this young cohort, microalbuminuria was already present in 9.9% of individuals, whereas the prevalence of diabetic retinopathy remained low at 0.3%. A multivariable logistic regression model (binomial distribution, logit link) was fitted to assess factors associated with microalbuminuria (*n* = 6012). Model fit was adequate (Pearson χ^2^/DF = 0.91). Sex, CSII use, age at diabetes onset, diabetes duration, and their interaction were not significantly associated with microalbuminuria. No migration background was associated with lower odds of microalbuminuria (OR 0.82, 95% CI: 0.68–0.99). Microalbuminuria increased with higher cardiovascular risk factor burden (Type III test: *p* = 0.0003). Compared to individuals without risk factors, ORs were 1.20 (95% CI: 0.98–1.46) for one risk factor, 1.45 (1.14–1.85) for two, 1.78 (1.22–2.59) for three and 2.68 (1.34–5.36) for four risk factors. In ordinal modelling, the odds ratio for microalbuminuria per additional cardiovascular risk factor was 1.22 (95% CI: 1.12–1.33; *p* < 0.001). In contrast, no significant associations with retinopathy were observed for any covariates or for cardiovascular risk factor burden (Type III test: *p* = 0.378; ordinal model *p* = 0.067).

## 4 Discussion

Adolescence and young adulthood represent a critical phase in the life course of individuals with T1DM, marked by developmental, psychological and health‐care transitions. In our cohort of 7298 emerging adults with paediatric‐onset T1DM, nearly half already exhibited at least one overt and inadequately controlled modifiable CVD risk factor, that is, poor glycaemic control, elevated BP > 130/80 mmHg, LDL cholesterol, obesity or smoking, and about one in five had two or more risk factors in addition to diabetes. These findings confirm population‐based data reporting high CVD risk and early diabetes‐related complications in adolescents and young adults with T1DM [30, 31, 32, 33, 34]. Recent analyses from the DPV registry indicate that the COVID‐19 pandemic further exacerbated cardiometabolic risk profiles in youth with T1DM [35]. The comparatively high cutoffs for glycaemic control (HbA1c > 9%), LDL cholesterol (> 130 mg/dL) and obesity were deliberately chosen to identify individuals with pronounced and actionable CVD risk. Lower thresholds, for example, HbA1c > 7%, affecting 79.8% of the entire cohort, or LDL > 100 mg/dL affecting 44.5%, would have classified the vast majority of our cohort as ‘high risk’, reflecting well‐known challenges in achieving guideline targets during the transition to adulthood. Accordingly, our approach likely underestimates the overall CVD risk burden while highlighting a high‐risk subgroup warranting urgent clinical attention. This underestimation is supported by the fact that individuals excluded due to incomplete documentation of risk factors exhibited an even higher CVD risk burden than those included in the final analytical cohort (Table S1).

The comparatively low prevalence of obesity in our cohort is likely related to the young age of the study population and is broadly consistent with age‐matched data from the general population, particularly among men [28, 36]. Here, poor glycaemic control was the most prevalent risk factor, consistent with international registry findings that achieving guideline‐recommended HbA1c targets remains particularly challenging during adolescence and young adulthood [17, 37, 38, 39]. Despite the broader use of CSII and AID, nearly one in five individuals using these advanced diabetes technologies continued to exhibit HbA1c levels > 9% (Figure S2c,d), underscoring that technology alone does not prevent severe glycaemic dysregulation in a high‐risk subgroup.

However, an even more concerning observation was the clustering of multiple CVD risk factors within the same individuals, which was already linked to microalbuminuria, representing the first microvascular complication at this young age. Even with a median diabetes duration of 8.5 years, a substantial proportion of young adults already exhibited at least one modifiable CVD risk factor, suggesting that cumulative metabolic and vascular exposure begins early in the disease course. Current guideline concepts incorporate diabetes duration into screening strategies, such as retinopathy screening after 5 years of disease duration and lower LDL treatment thresholds with longer diabetes duration.

These findings align with data from the International Childhood Cardiovascular Cohort (i3C) Consortium, showing that five traditional CVD risk factors, tha is, BMI, BP, triglycerides, total cholesterol and smoking, predict adult CVD events and premature CVD death before the age of 60 years [40]. Importantly, this study found an increased risk even among those in the high‐normal or high‐acceptable categories for the BMI, systolic blood pressure and triglycerides. Similarly, several vascular imaging studies, as well as autopsy data from the Bogalusa heart study, demonstrated an association between the number of CVD risk factors and the extent of asymptomatic atherosclerotic lesions in the aorta and coronary arteries of children and young adults [41, 42, 43]. Together, these findings indicate a synergistic nature of CVD risk, as cumulative exposure to hyperglycaemia, hypertension, dyslipidaemia and obesity accelerates vascular injury long before overt CVD disease becomes apparent. Triglyceride‐related markers have recently gained increased attention in CVD risk stratification in adults with T1DM, however, were not included in this analysis due to non‐standardised, predominantly non‐fasting measurements in the registry setting [44], whereas LDL‐cholesterol is minimally affected by fasting status [45].

Longitudinal i3C data and related cohort studies indicate that resolution of obesity, hypertension, or dyslipidaemia is associated with a substantial attenuation of later atherosclerotic and cardiometabolic risk [46, 47, 48], although this evidence is largely based on adult risk factor status rather than hard CV outcomes. It does not specifically address populations with T1DM nor the mechanisms underlying risk factor resolution in adulthood.

Our findings should be interpreted as reflecting CVD risk burden and treatment gaps, rather than the effectiveness of pharmacological interventions. Importantly, among individuals with clear treatment indications, only 19.8% (26/131) with arterial hypertension > 140/90 mmHg and only 9.9% (*n* = 101/1023) of those with LDL cholesterol > 130 mg/dL received targeted pharmacotherapy, indicating clinically relevant treatment inertia despite clear guideline‐based indications in this vulnerable age group. Compared with 2006 DPV data (0.4% and 0.8%, respectively) this represents a relevant improvement, yet considerable undertreatment persists [32]. Notably, individuals on lipid‐lowering therapy in our study had significantly higher LDL and total cholesterol levels than untreated peers, suggesting medication was often initiated only after marked dyslipidaemia and that intensification towards target levels remains insufficient.

Heterogeneity among guidelines likely contributes to this inertia. The International Society for Paediatric and Adolescent Diabetes (ISPAD) recommends statin initiation from age 10 years if LDL > 130 mg/dL persists despite lifestyle intervention [49], whereas the American Diabetes Association (ADA) and German S3 Guideline for Paediatric Diabetes Care (AWMF 057‐016) suggest pharmacotherapy only at LDL > 160 mg/dL or in the presence of additional risk factors [50]. In line with adult guidelines (German S3 National Disease Management Guideline on Hypertension, AWMF nvl‐009 and German S3 Guideline for Diabetes Care, AWMF 057–013), treatment for hypertension is generally initiated at sustained BP > 130/80 mmHg in adolescents or > 140/90 mmHg in adults. Our analysis, therefore, focused on the latter threshold, which is consistently used in clinical practice.

Of obese participants, 10% received metformin, most likely reflecting individualised off‐label use to address insulin resistance or clustered cardiometabolic risk factors. Patients on antihypertensive or lipid‐lowering therapy were also more likely to receive metformin, indicating that those with a metabolic‐syndrome phenotype more frequently undergo multifaceted pharmacotherapy. As the primary aim was to characterise clinically relevant treatment gaps, potential survival and treatment bias are unlikely to affect the main conclusions. These results reveal a substantial gap between real‐world care and guideline recommendations. Given robust evidence that tighter control of HbA1c, systolic BP and LDL cholesterol reduces CVD morbidity and mortality in T1DM [11, 51], the low implementation rates are concerning.

The recently published *Lancet Commission on rethinking coronary artery disease* calls for a paradigm shift from ischemia‐centred strategies towards lifelong atheroma prevention, estimating that such an approach could prevent up to 8.7 million deaths annually [52]. This preventive perspective is directly relevant to young individuals with T1DM, whose lifetime exposure to metabolic and hemodynamic stress is exceptionally high. Structured prevention strategies are essential during adolescence and the transition to adult care, a period known for deteriorating glycaemic control, reduced medical engagement and delayed treatment intensification [14, 15]. Although current guidelines recommend regular screening for CVD risk factors starting in adolescence, our findings suggest that screening alone is insufficient; the critical gap lies in translating detection into effective, sustained treatment. Beyond established CVD risk factors, protective lifestyle‐related factors, including regular physical activity as incorporated in the ST1RE [53], and oxidative stress–modulating mechanisms may contribute to CVD risk reduction in T1DM, whereas experimental and translational studies have shown that dysregulated lipid metabolism and endothelial dysfunction play a key role in early vascular damage [54].

Strengths of this study include the large, representative sample of young adults with paediatric‐onset T1DM, drawn from a well‐established, nationwide registry with high coverage and standardised data collection. The analysis also provides novel insights into the co‐occurrence of modifiable risk factors and their link to early vascular complications. However, the cross‐sectional design precludes causal inference. Another limitation is that registry‐based data may be subject to documentation heterogeneity. Although clinical parameters were assessed over a multi‐year period (2020–2023), median values were used to enhance robustness. Treatment data reflect only the most recently documented therapeutic status and may not capture long‐term treatment continuity or adherence. The lack of statistical significance in the linear model for retinopathy is likely attributable to the very low prevalence of retinopathy at this early stage of the disease course (0.3%), limiting statistical power to detect associations. In conclusion, adolescents and young adults with paediatric‐onset T1DM display a high burden of pronounced and clinically actionable, modifiable CVD risk factors and significant treatment gaps. The accumulation of multiple risk factors is associated with early microvascular damage, underlining the urgency of early, comprehensive and sustained CVD prevention. Proactive screening and timely treatment in paediatric diabetes care are crucial to avoid therapeutic inertia and to improve long‐term CVD outcomes. Paediatric providers play a key role in this context and should not wait for adult care providers to take action.

## Author Contributions

A.W. and R.W.H. conceived the study and discussed the structure of the manuscript. A.W. and C.R. interpreted the data and wrote the manuscript with help from R.W.H. All authors contributed to the study design and critically read and revised the manuscript.

## Funding

The study was financially supported by the Federal Ministry of Education and Research within the German Centre for Diabetes Research (DZD, FKZ 82DZD14E03). The German Diabetes Association (DDG), the Robert Koch Institute, Germany, provided further financial support. Sponsors were not involved in data acquisition or analysis. Funders were not involved in the analysis and interpretation of data, the writing of the report, or the decision to submit the article for publication.

## Ethics Statement

The DPV initiative and the analysis of anonymised data related to quality of care are approved by the ethics committee of the University of Ulm (reference no. 314/21) and by local review boards of the participating centres.

## Conflicts of Interest

The authors declare no conflicts of interest.

## Supporting information


**Figure S1:** dom70610‐sup‐0001‐Figures.pdf.
**Figure S1:** Flowchart of patient selection.
**Figure S2a–d:** Prevalence of cardiovascular risk factors stratified by migration background (a), age at diabetes onset (b) and treatment (c and d). Prevalence of poor glycaemic control (HbA1c > 9%), elevated blood pressure (> 130/80 mmHg), elevated LDL cholesterol (> 130 mg/dL), obesity (BMI > 30 kg/m^2^) and current smoking among young adults with T1DM stratified by migration background (a), prepubertal onset (< 11 years in girls, < 12 years in boys) versus pubertal/postpubertal onset (≥ 11 or ≥ 12 years, respectively) (b); and treatment modalities CSII (*n* = 4315) vs. MDI (*n* = 2983) (c) and AID (automated insulin delivery, *n* = 1693) vs. CSII/MDI (d; *n* = 5605); *** *p* < 0.001; * *p* < 0.05.
**Figure S3:** List of participating DPV centres (alphabetical order).

**Table S1:** Comparison of included individuals with complete documentation of all five predefined CV risk factors and excluded cases with incomplete risk factor documentation. Data are presented as medians with interquartile ranges (Q1–Q3) or percentages. Group differences were assessed using Wilcoxon tests for continuous variables and chi‐square tests for categorical variables; corresponding *p*‐values are shown.

## Data Availability

Aggregated datasets used and/or analysed during the current study are available from the corresponding author on reasonable request. Due to protection of patient privacy, patient‐level data cannot be shared, but joint analysis projects are possible.

## References

[1] G. D. Ogle , F. Wang , G. A. Gregory , and J. Maniam , “Type 1 Diabetes Estimates in Children and Adults,” International Diabetes Federation (2022).

[2] G. A. Gregory , T. I. G. Robinson , S. E. Linklater , et al., “Global Incidence, Prevalence, and Mortality of Type 1 Diabetes in 2021 With Projection to 2040: A Modelling Study,” Lancet Diabetes and Endocrinology 10, no. 10 (2022): 741–760. 36113507 10.1016/S2213-8587(22)00218-2

[3] M. Lind , A. M. Svensson , and A. Rosengren , “Glycemic Control and Excess Mortality in Type 1 Diabetes,” New England Journal of Medicine 372, no. 9 (2015): 880–881. 10.1056/NEJMc1415677 25714168

[4] A. Rawshani , N. Sattar , S. Franzen , et al., “Excess Mortality and Cardiovascular Disease in Young Adults With Type 1 Diabetes in Relation to Age at Onset: A Nationwide, Register‐Based Cohort Study,” Lancet 392, no. 10146 (2018): 477–486. 30129464 10.1016/S0140-6736(18)31506-X PMC6828554

[5] V. Harjutsalo , D. Pongrac Barlovic , and P. H. Groop , “Long‐Term Population‐Based Trends in the Incidence of Cardiovascular Disease in Individuals With Type 1 Diabetes From Finland: A Retrospective, Nationwide, Cohort Study,” Lancet Diabetes and Endocrinology 9, no. 9 (2021): 575–585. 34303414 10.1016/S2213-8587(21)00172-8

[6] S. J. Livingstone , H. C. Looker , E. J. Hothersall , et al., “Risk of Cardiovascular Disease and Total Mortality in Adults With Type 1 Diabetes: Scottish Registry Linkage Study,” PLoS Medicine 9, no. 10 (2012): e1001321. 23055834 10.1371/journal.pmed.1001321 PMC3462745

[7] S. J. Livingstone , D. Levin , H. C. Looker , et al., “Estimated Life Expectancy in a Scottish Cohort With Type 1 Diabetes, 2008–2010,” JAMA 313, no. 1 (2015): 37–44. 25562264 10.1001/jama.2014.16425 PMC4426486

[8] M. Arffman , P. Hakkarainen , I. Keskimaki , T. Oksanen , and R. Sund , “Long‐Term and Recent Trends in Survival and Life Expectancy for People With Type 1 Diabetes in Finland,” Diabetes Research and Clinical Practice 198 (2023): 110580. 36804193 10.1016/j.diabres.2023.110580

[9] R. G. Miller , H. D. Mahajan , T. Costacou , A. Sekikawa , S. J. Anderson , and T. J. Orchard , “A Contemporary Estimate of Total Mortality and Cardiovascular Disease Risk in Young Adults With Type 1 Diabetes: The Pittsburgh Epidemiology of Diabetes Complications Study,” Diabetes Care 39, no. 12 (2016): 2296–2303. 27654986 10.2337/dc16-1162 PMC5127232

[10] M. Lind , A. M. Svensson , M. Kosiborod , et al., “Glycemic Control and Excess Mortality in Type 1 Diabetes,” New England Journal of Medicine 371, no. 21 (2014): 1972–1982. 25409370 10.1056/NEJMoa1408214

[11] A. Rawshani , A. Rawshani , N. Sattar , et al., “Risk Factors, Mortality, and Cardiovascular Outcomes in Patients With Type 2 Diabetes,” Circulation 139, no. 16 (2019): 1900–1912. 30798638 10.1161/CIRCULATIONAHA.118.037454

[12] Z. Liu , H. Wang , Z. Yang , Y. Lu , and C. Zou , “Causal Associations Between Type 1 Diabetes Mellitus and Cardiovascular Diseases: A Mendelian Randomization Study,” Cardiovascular Diabetology 22, no. 1 (2023): 236. 37659996 10.1186/s12933-023-01974-6 PMC10475187

[13] C. Hero , A. Rawshani , A. M. Svensson , et al., “Association Between Use of Lipid‐Lowering Therapy and Cardiovascular Diseases and Death in Individuals With Type 1 Diabetes,” Diabetes Care 39, no. 6 (2016): 996–1003. 27208327 10.2337/dc15-2450

[14] A. Peters , L. Laffel , and American Diabetes Association Transitions Working G , “Diabetes Care for Emerging Adults: Recommendations for Transition From Pediatric to Adult Diabetes Care Systems: A Position Statement of the American Diabetes Association, With Representation by the American College of Osteopathic Family Physicians, the American Academy of Pediatrics, the American Association of Clinical Endocrinologists, the American Osteopathic Association, the Centers for Disease Control and Prevention, Children With Diabetes, the Endocrine Society, the International Society for Pediatric and Adolescent Diabetes, Juvenile Diabetes Research Foundation International, the National Diabetes Education Program, and the Pediatric Endocrine Society (Formerly Lawson Wilkins Pediatric Endocrine Society),” Diabetes Care 34, no. 11 (2011): 2477–2485. 22025785 10.2337/dc11-1723 PMC3198284

[15] R. P. D'Amico , T. M. Pian , and E. O. Buschur , “Transition From Pediatric to Adult Care for Individuals With Type 1 Diabetes: Opportunities and Challenges,” Endocrine Practice 29, no. 4 (2023): 279–285. 36528273 10.1016/j.eprac.2022.12.006

[16] M. A. Clements , N. C. Foster , D. M. Maahs , et al., “Hemoglobin A1c (HbA1c) Changes Over Time Among Adolescent and Young Adult Participants in the T1D Exchange Clinic Registry,” Pediatric Diabetes 17, no. 5 (2016): 327–336. 26153338 10.1111/pedi.12295

[17] N. C. Foster , R. W. Beck , K. M. Miller , et al., “State of Type 1 Diabetes Management and Outcomes From the T1D Exchange in 2016‐2018,” Diabetes Technology and Therapeutics 21, no. 2 (2019): 66–72. 30657336 10.1089/dia.2018.0384 PMC7061293

[18] L. M. Laffel , L. Vangsness , A. Connell , A. Goebel‐Fabbri , D. Butler , and B. J. Anderson , “Impact of Ambulatory, Family‐Focused Teamwork Intervention on Glycemic Control in Youth With Type 1 Diabetes,” Journal of Pediatrics 142, no. 4 (2003): 409–416. 12712059 10.1067/mpd.2003.138

[19] D. S. Lotstein , M. Seid , G. Klingensmith , et al., “Transition From Pediatric to Adult Care for Youth Diagnosed With Type 1 Diabetes in Adolescence,” Pediatrics 131, no. 4 (2013): e1062–e1070. 23530167 10.1542/peds.2012-1450 PMC4535025

[20] T. M. Kapellen , S. Muther , A. Schwandt , et al., “Transition to Adult Diabetes Care in Germany‐High Risk for Acute Complications and Declining Metabolic Control During the Transition Phase,” Pediatric Diabetes 19 (2018): 1094–1099. 10.1111/pedi.12687 29691964

[21] S. T. Chiesa and M. L. Marcovecchio , “Preventing Cardiovascular Complications in Type 1 Diabetes: The Need for a Lifetime Approach,” Frontiers in Pediatrics 9 (2021): 696499. 34178905 10.3389/fped.2021.696499 PMC8219852

[22] K. N. L.‐J. Karmali and M. Donald , “Achieving and Maintaining Cardiovascular Health Across the Lifespan,” Current Epidemiology Reports 1 (2014): 75–81.

[23] A. Dost , S. Bechtold , K. Fink , et al., “2017 American Academy of Pediatrics Clinical Practice Guideline: Impact on Prevalence of Arterial Hypertension in Children and Adolescents With Type 1 Diabetes,” Diabetes Care 43, no. 6 (2020): 1311–1318. 32229598 10.2337/dc19-2022

[24] A. K. Kershnar , S. R. Daniels , G. Imperatore , et al., “Lipid Abnormalities Are Prevalent in Youth With Type 1 and Type 2 Diabetes: The SEARCH for Diabetes in Youth Study,” Journal of Pediatrics 149, no. 3 (2006): 314–319. 16939739 10.1016/j.jpeds.2006.04.065

[25] S. K. Lyons , C. T. Boyle , D. J. DeSalvo , et al., “Dyslipidaemia and Statin Use in Individuals Aged 10 to < 40 Years in the T1D Exchange Clinic Registry,” Diabetes, Obesity and Metabolism 21, no. 1 (2019): 170–172. 10.1111/dom.13475 PMC7059116 30039636

[26] D. M. Maahs , S. R. Daniels , S. D. de Ferranti , et al., “Cardiovascular Disease Risk Factors in Youth With Diabetes Mellitus: A Scientific Statement From the American Heart Association,” Circulation 130, no. 17 (2014): 1532–1558. 25170098 10.1161/CIR.0000000000000094

[27] B. L. Rodriguez , D. Dabelea , A. D. Liese , et al., “Prevalence and Correlates of Elevated Blood Pressure in Youth With Diabetes Mellitus: The SEARCH for Diabetes in Youth Study,” Journal of Pediatrics 157, no. 2 (2010): 245–251. 20394942 10.1016/j.jpeds.2010.02.021

[28] A. Welters , S. R. Tittel , K. Laubner , et al., “Long‐Term Trends of BMI and Cardiometabolic Risk Factors Among Adults With Type 1 Diabetes: An Observational Study From the German/Austrian DPV Registry,” Diabetes Research and Clinical Practice 178 (2021): 108973. 34302914 10.1016/j.diabres.2021.108973

[29] J. Rosenbauer , A. Neu , U. Rothe , J. Seufert , and R. W. Holl , “Types of Diabetes Are Not Limited to Age Groups: Type 1 Diabetes in Adults and Type 2 Diabetes in Children and Adolescents,” Journal of Health Monitoring 4, no. 2 (2019): 29–49. 10.25646/5987 PMC8822252 35146246

[30] I. Drozd , J. Weiskorn , K. Lange , et al., “Prevalence of LDL‐Hypercholesterolemia and Other Cardiovascular Risk Factors in Young People With Type 1 Diabetes,” Journal of Clinical Lipidology 17, no. 4 (2023): 483–490. 37258406 10.1016/j.jacl.2023.05.097

[31] K. A. Sauder , J. M. Stafford , E. J. Mayer‐Davis , et al., “Co‐Occurrence of Early Diabetes‐Related Complications in Adolescents and Young Adults With Type 1 Diabetes: An Observational Cohort Study,” Lancet Child Adolesc Health 3, no. 1 (2019): 35–43. 30409691 10.1016/S2352-4642(18)30309-2 PMC6295346

[32] K. O. Schwab , J. Doerfer , W. Hecker , et al., “Spectrum and Prevalence of Atherogenic Risk Factors in 27,358 Children, Adolescents, and Young Adults With Type 1 Diabetes: Cross‐Sectional Data From the German Diabetes Documentation and Quality Management System (DPV),” Diabetes Care 29, no. 2 (2006): 218–225. 16443863 10.2337/diacare.29.02.06.dc05-0724

[33] K. O. Schwab , J. Doerfer , W. Marg , E. Schober , R. W. Holl , and Initiative DPVS , “Characterization of 33 488 Children and Adolescents With Type 1 Diabetes Based on the Gender‐Specific Increase of Cardiovascular Risk Factors,” Pediatric Diabetes 11, no. 5 (2010): 357–363. 20624248 10.1111/j.1399-5448.2010.00665.x

[34] C. Steigleder‐Schweiger , B. Rami‐Merhar , T. Waldhor , et al., “Prevalence of Cardiovascular Risk Factors in Children and Adolescents With Type 1 Diabetes in Austria,” European Journal of Pediatrics 171, no. 8 (2012): 1193–1202. 22422191 10.1007/s00431-012-1704-x

[35] A. J. Eckert , S. Linke , K. O. Schwab , et al., “Changes in Cardiovascular Risk Factors Among Children and Young Adults With Type 1 Diabetes During the COVID‐19 Pandemic Compared to Previous Years‐Results From the German DPV Registry,” Journal of Diabetes 15, no. 1 (2023): 15–26. 36621521 10.1111/1753-0407.13340 PMC9870744

[36] A. Schienkiewitz , R. Kuhnert , M. Blume , and G. B. M. Mensink , “Overweight and Obesity Among Adults in Germany ‐ Results From GEDA 2019/2020‐EHIS,” Journal of Health Monitoring 7, no. 3 (2022): 21–28. 10.25646/10293 PMC9520353 36188152

[37] D. J. DeSalvo , K. M. Miller , J. M. Hermann , et al., “Continuous Glucose Monitoring and Glycemic Control Among Youth With Type 1 Diabetes: International Comparison From the T1D Exchange and DPV Initiative,” Pediatric Diabetes 19, no. 7 (2018): 1271–1275. 29923262 10.1111/pedi.12711 PMC6175652

[38] F. S. Malik , K. A. Sauder , S. Isom , et al., “Trends in Glycemic Control Among Youth and Young Adults With Diabetes: The SEARCH for Diabetes in Youth Study,” Diabetes Care 45, no. 2 (2022): 285–294. 34995346 10.2337/dc21-0507 PMC8914430

[39] A. T. Zimmermann , S. Lanzinger , S. J. Kummernes , et al., “Treatment Regimens and Glycaemic Outcomes in More Than 100 000 Children With Type 1 Diabetes (2013‐22): A Longitudinal Analysis of Data From Paediatric Diabetes Registries,” Lancet Diabetes and Endocrinology 13, no. 1 (2025): 47–56. 39622257 10.1016/S2213-8587(24)00279-1

[40] D. R. Jacobs, Jr. , J. G. Woo , A. R. Sinaiko , et al., “Childhood Cardiovascular Risk Factors and Adult Cardiovascular Events,” New England Journal of Medicine 386, no. 20 (2022): 1877–1888. 35373933 10.1056/NEJMoa2109191 PMC9563825

[41] G. S. Berenson , S. R. Srinivasan , W. Bao , W. P. Newman , R. E. Tracy , and W. A. Wattigney , “Association Between Multiple Cardiovascular Risk Factors and Atherosclerosis in Children and Young Adults. The Bogalusa Heart Study,” New England Journal of Medicine 338, no. 23 (1998): 1650–1656. 9614255 10.1056/NEJM199806043382302

[42] M. F. Fusaro , J. L. Zanini , and I. N. Silva , “Increased Carotid Intima‐Media Thickness in Brazilian Adolescents With Type 1 Diabetes Mellitus,” Diabetology and Metabolic Syndrome 8 (2016): 74. 27895720 10.1186/s13098-016-0190-0 PMC5106830

[43] O. T. Raitakari , M. Juonala , M. Kahonen , et al., “Cardiovascular Risk Factors in Childhood and Carotid Artery Intima‐Media Thickness in Adulthood: The Cardiovascular Risk in Young Finns Study,” Journal of the American Medical Association 290, no. 17 (2003): 2277–2283. 14600186 10.1001/jama.290.17.2277

[44] R. Oh , S. Kim , S. H. Park , et al., “Elevated Triglyceride‐Glucose Index Is a Risk Factor for Cardiovascular Events in Adults With Type 1 Diabetes: A Cohort Study,” Cardiovascular Diabetology 24, no. 1 (2025): 150. 40176060 10.1186/s12933-025-02712-w PMC11966936

[45] B. G. Nordestgaard , A. Langsted , S. Mora , et al., “Fasting Is Not Routinely Required for Determination of a Lipid Profile: Clinical and Laboratory Implications Including Flagging at Desirable Concentration Cut‐Points—A Joint Consensus Statement From the European Atherosclerosis Society and European Federation of Clinical Chemistry and Laboratory Medicine,” European Heart Journal 37, no. 25 (2016): 1944–1958. 27122601 10.1093/eurheartj/ehw152 PMC4929379

[46] J. Juhola , C. G. Magnussen , G. S. Berenson , et al., “Combined Effects of Child and Adult Elevated Blood Pressure on Subclinical Atherosclerosis: The International Childhood Cardiovascular Cohort Consortium,” Circulation 128, no. 3 (2013): 217–224. 23780579 10.1161/CIRCULATIONAHA.113.001614 PMC3875837

[47] M. Juonala , C. G. Magnussen , G. S. Berenson , et al., “Childhood Adiposity, Adult Adiposity, and Cardiovascular Risk Factors,” New England Journal of Medicine 365, no. 20 (2011): 1876–1885. 22087679 10.1056/NEJMoa1010112

[48] C. G. Magnussen , J. Koskinen , M. Juonala , et al., “A Diagnosis of the Metabolic Syndrome in Youth That Resolves by Adult Life Is Associated With a Normalization of High Carotid Intima‐Media Thickness and Type 2 Diabetes Mellitus Risk: The Bogalusa Heart and Cardiovascular Risk in Young Finns Studies,” Journal of the American College of Cardiology 60, no. 17 (2012): 1631–1639. 23021330 10.1016/j.jacc.2012.05.056

[49] P. Bjornstad , A. Dart , K. C. Donaghue , et al., “ISPAD Clinical Practice Consensus Guidelines 2022: Microvascular and Macrovascular Complications in Children and Adolescents With Diabetes,” Pediatric Diabetes 23, no. 8 (2022): 1432–1450. 36537531 10.1111/pedi.13444

[50] American Diabetes Association Professional Practice C , “14. Children and Adolescents: Standards of Care in Diabetes‐2025,” Diabetes Care 48, no. 1 Suppl 1 (2025): S283–S305. 39651980 10.2337/dc25-S014 PMC11635046

[51] A. Rawshani , A. Rawshani , S. Franzen , et al., “Range of Risk Factor Levels: Control, Mortality, and Cardiovascular Outcomes in Type 1 Diabetes Mellitus,” Circulation 135, no. 16 (2017): 1522–1531. 28416524 10.1161/CIRCULATIONAHA.116.025961 PMC5400410

[52] S. Zaman , J. H. Wasfy , V. Kapil , et al., “The Lancet Commission on Rethinking Coronary Artery Disease: Moving From Ischaemia to Atheroma,” Lancet 405, no. 10486 (2025): 1264–1312. 40179933 10.1016/S0140-6736(25)00055-8 PMC12315672

[53] D. Vistisen , G. S. Andersen , C. S. Hansen , et al., “Prediction of First Cardiovascular Disease Event in Type 1 Diabetes Mellitus: The Steno Type 1 Risk Engine,” Circulation 133, no. 11 (2016): 1058–1066. 26888765 10.1161/CIRCULATIONAHA.115.018844

[54] K. Rehman , K. Haider , K. Jabeen , and M. S. H. Akash , “Current Perspectives of Oleic Acid: Regulation of Molecular Pathways in Mitochondrial and Endothelial Functioning Against Insulin Resistance and Diabetes,” Reviews in Endocrine and Metabolic Disorders 21, no. 4 (2020): 631–643. 32125563 10.1007/s11154-020-09549-6

