# Supplementary material for: Highly Uncontrolled Cardiovascular Risk in Emerging Adults With Paediatric‐Onset Type 1 Diabetes—A Cross‐Sectional Analysis From the Diabetes Prospective Follow‐Up Registry DPV
Source: Diabetes Obes Metab. 2026 Mar 16;28(5):4194–204. doi: 10.1111/dom.70610 (PMC13071203; doi:10.1111/dom.70610)
Supplement: Supplementary file 1 — Figure S1: dom70610‐sup‐0001‐Figures.pdf. Figure S1: Flowchart of patient selection. Figure S2a–d: Prevalence of cardiovascular risk factors stratified by migration background (a), age at diabetes onset (b) and treatment (c and d). Prevalence of poor glycaemic control (HbA1c > 9%), elevated blood pressure (> 130/80 mmHg), elevated LDL cholesterol (> 130 mg/dL), obesity (BMI > 30 kg/m2) and current smoking among young adults with T1DM stratified by migration background (a), prepubertal onset (< 11 years in girls, < 12 years in boys) versus pubertal/postpubertal onset (≥ 11 or ≥ 12 years, respectively) (b); and treatment modalities CSII (n = 4315) vs. MDI (n = 2983) (c) and AID (automated insulin delivery, n = 1693) vs. CSII/MDI (d; n = 5605); *** p < 0.001; * p < 0.05. Figure S3: List of participating DPV centres (alphabetical order). [file DOM-28-4194-s001.pdf]

Supp. Fig. 1 Flowchart of patient selection

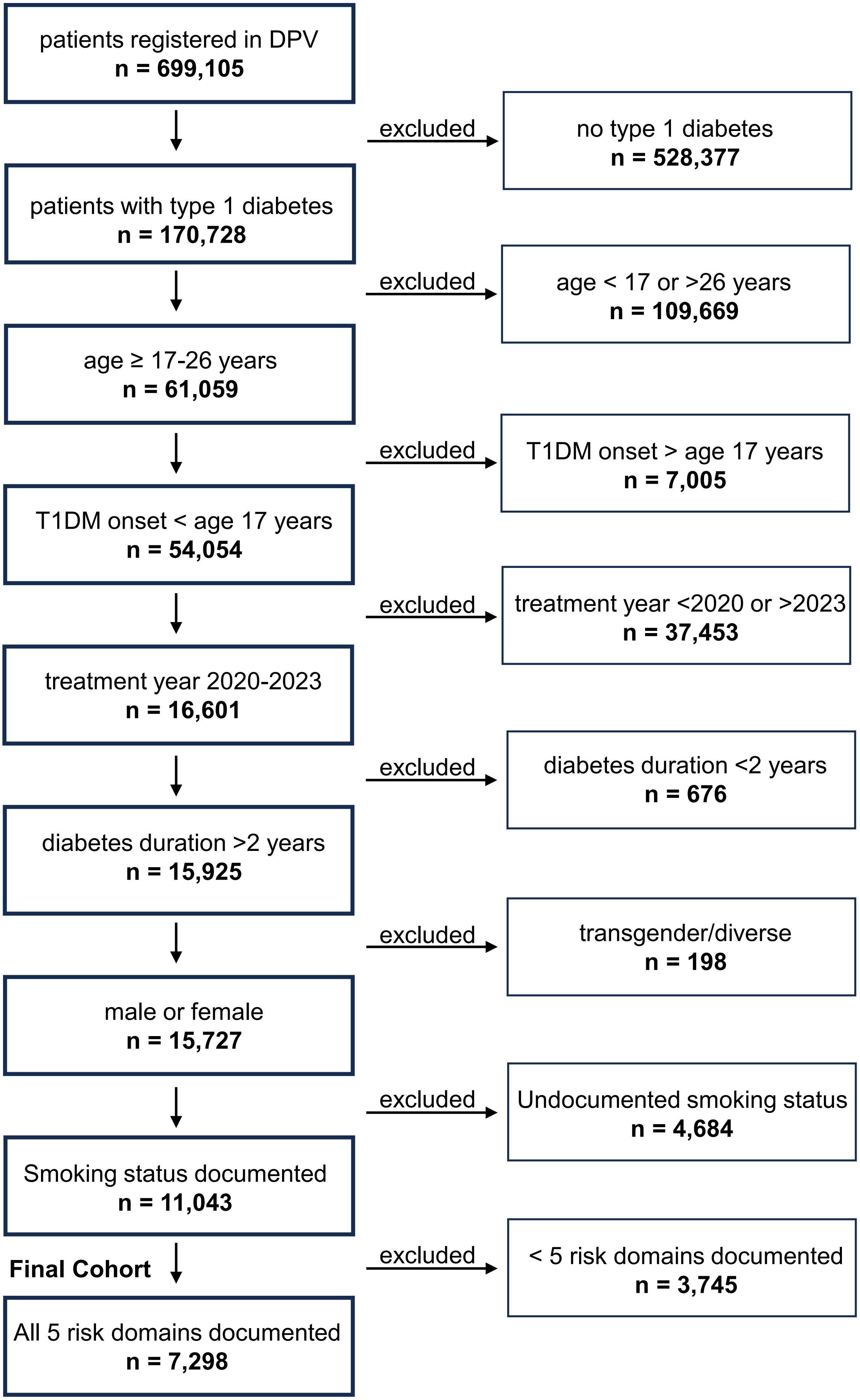

Supp. Fig. 2a and b

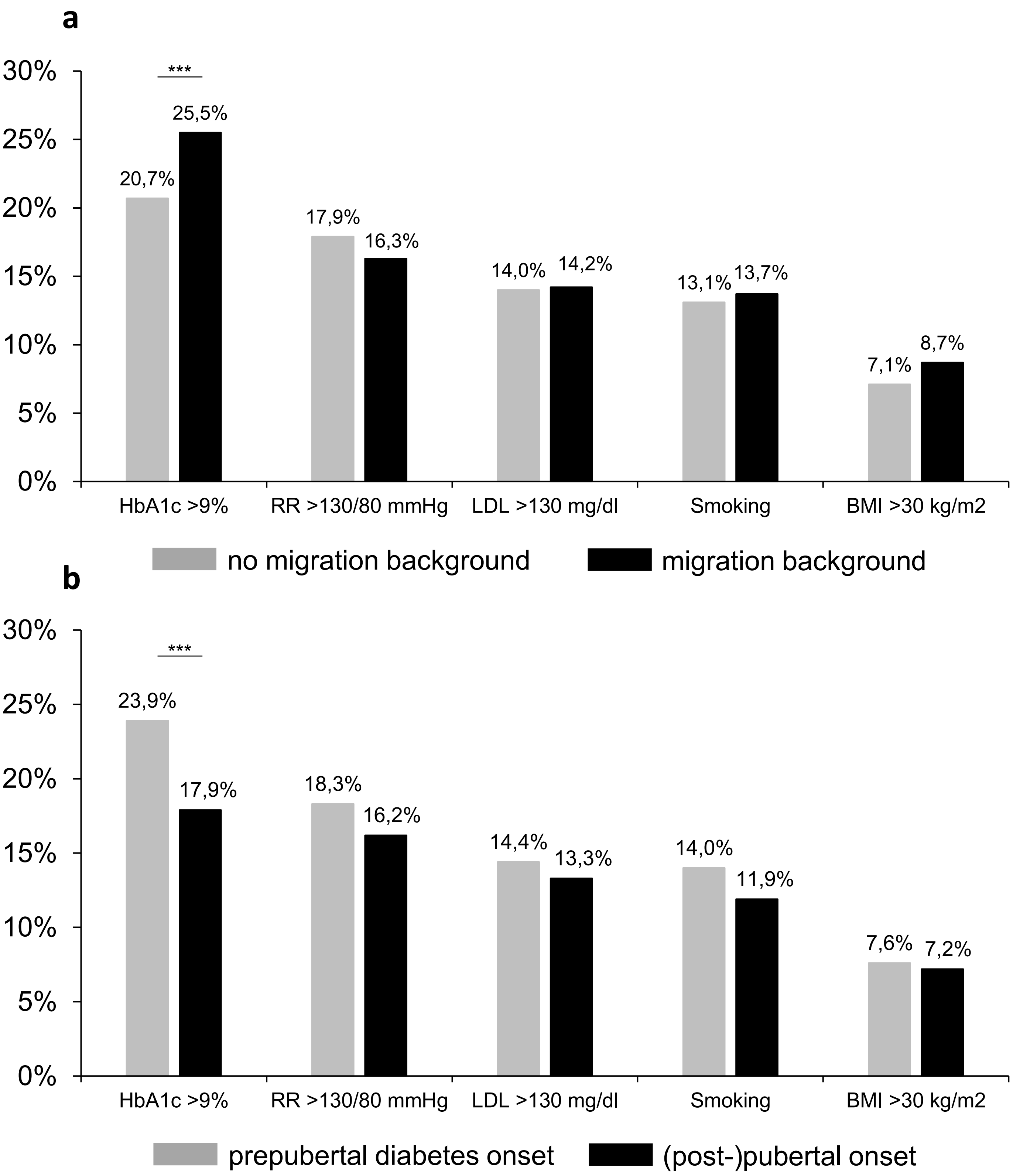

Supp. Fig. 2c and d

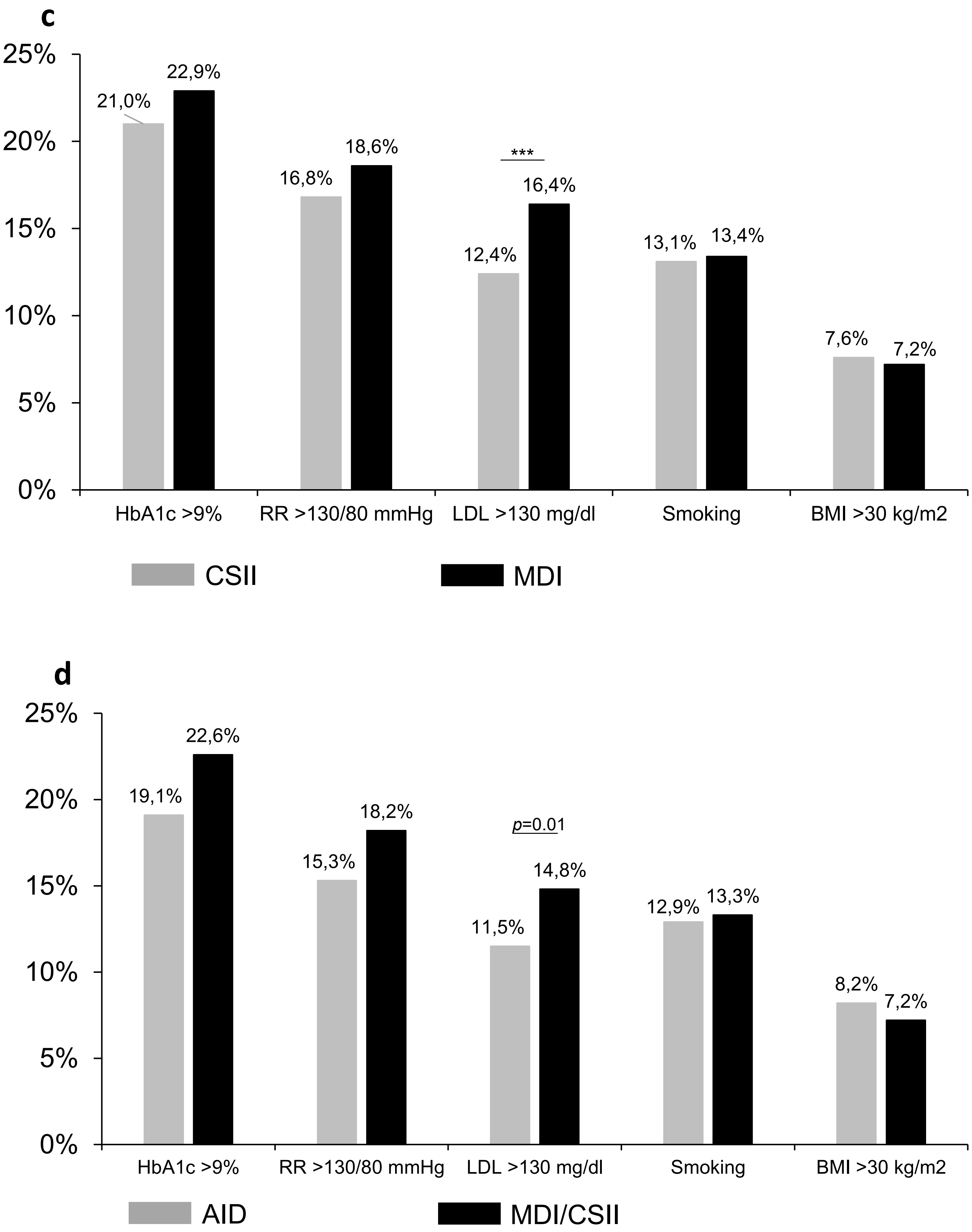

Supp. Fig. 3

List of participating DPV centres (alphabetical order)

Aachen - Innere RWTH
Aachen - Uni-Kinderklinik RWTH
Aalen Kinderklinik
Ahlen St. Franziskus Kinderklinik
Altötting Kinderklinik Zentrum Inn-Salzach
Amberg Kinderklinik St. Marien
Arnsberg-Hüsten Karolinenhosp. Kinderabteilung
Asbach Kamillus-Klinik Innere
Aue Helios Kinderklink
Augsburg IV. Med. Uni-Klinik
Augsburg Josefinum Kinderklinik
Augsburg Uni-Kinderklinik
Aurich Kinderklinik
Bad Aibling Internist. Praxis
Bad Hersfeld Innere
Bad Hersfeld Kinderklinik
Bad Kreuznach Diakonie Kikli
Bad Kreuznach-Viktoriastift
Bad Kösen Median Kinderklinik
Bad Mergentheim - Diabetesfachklinik
Bad Mergentheim - Kinderdiabetologische Praxis
Bad Oeynhausen Herz-und Diabeteszentrum NRW
Bad Orb Spessart Klinik
Bad Reichenhall Kreisklinik Innere Med.
Bad Salzungen Kinderklinik
Basel Uni-Kinderspital beider Basel (UKBB)

Bautzen Oberlausitz KK
Bayreuth Innere Medizin
Berchtesgaden CJD
Berchtesgaden CJD-Beruf.REHA
Berlin DRK-Kliniken Mitte Innere
Berlin DRK-Kliniken Pädiatrie
Berlin Klinik St. Hedwig Innere
Berlin Lichtenberg - Kinderklinik
Berlin Oskar Zieten Krankenhaus Innere
Berlin Parkklinik Weissensee
Berlin Schlosspark-Klinik Innere
Berlin Virchow-Kinderklinik
Berlin Vivantes Hellersdorf Innere
Bern Inselspital Kinderklinik
Bern Universitätsklinik für Diabetologie und Endokrinologie
Bielefeld Kinderklinik Gilead
Bielefeld Pädiatrisches Forum
Bocholt Kinderklinik
Bochum Universitätskinderklinik St. Josef
Bodnegg - MVZ Wollmarshöhe
Bonn Schwerpunktpraxis
Bonn Uni-Kinderklinik
Bottrop Knappschaftskrankenhaus Innere
Braunschweig Kinderarztpraxis
Bremen - Kinderklinik Nord
Bremen Eltern-Kind-Zentrum, Klinikum Mitte

Bremerhaven Kinderklinik
Bruchweiler Edelsteinklinik Kinder-Reha
Böblingen Kinderklinik
Celle Kinderarztpraxis
Celle Klinik für Kinder- und Jugendmedizin
Chemnitz Kinderklinik
Coburg Innere Medizin
Coburg Kinderklinik
Coesfeld Kinderklinik
Coesfeld/Dülmen Innere Med.
Darmstadt Innere Medizin
Darmstadt Kinderklinik Prinz. Margaret
Datteln Vestische Kinderklinik
Deggendorf Medizinische Klinik II
Deggendorf Pädiatrie-Praxis
Delmenhorst JHD Kinderklinik
Dessau Kinderklinik
Dessau amb. Kinderarztzentrum
Dornbirn Kinderklinik
Dortmund Johannes Hospital
Dortmund Kinderklinik
Dortmund Knappschaftskrankenhaus Innere
Dortmund Medizinische Kliniken Nord
Dortmund-St. Josefshospital Innere
Dortmund-West Innere
Dresden Neustadt Kinderklinik

Dresden Uni-Kinderklinik
Duisburg Homberg Helios Rhein-Ruhr Kliniken GmbH
Duisburg Sana Kinderklinik
Duisburg St. Anna Innere Helios Rhein-Ruhr Kliniken GmbH
Duisburg-St.Johannes Helios
Düren-Birkesdorf Kinderklinik
Düsseldorf Uni-Kinderklinik
Eberswalde Werner Forßmann Klinikum
Eckernförde Gem.-Prax
Eisleben Lutherstadt Helios-Klinik
Erfurt Kinderklinik
Erlangen Uni Innere Medizin
Erlangen Uni-Kinderklinik
Essen Diabetes-SPP
Essen Diabetes-Schwerpunktpraxis
Essen Elisabeth Kinderklinik
Essen Kinderarztpraxis
Esslingen Klinik für Kinder und Jugendliche
Esslingen Schwerpunktpraxis
Feldkirch Kinderklinik
Filderstadt Kinderklinik
Frankenthal Kinderarztpraxis
Frankfurt Diabeteszentrum Rhein-Main-Erwachsenendiabetologie (Bürgerhospital)
Frankfurt Diabeteszentrum Rhein-Main-pädiat. Diabetologie (Clementine-Hospital)
Frankfurt Uni-Kinderklinik
Frankfurt-Höchst, Städtische Kinderklinik

Frankfurt-Sachsenhausen Innere
Freiburg St. Josef Kinderklinik
Freiburg Uni Innere
Freiburg Uni-Kinderklinik
Freudenstadt Kinderklinik
Fulda Kinderklinik
Fürth Kinderklinik
Gaissach Fachklinik der Deutschen Rentenversicherung Bayern Süd
Garmisch-Partenkirchen Kinderklinik
Garmisch-Partenkirchen Klinikum Pädiatrie
Geislingen Klinik Helfenstein Innere
Gelnhausen Kinderklinik
Gelsenkirchen Kinderklinik Marienhospital
Gera Kinderklinik
Gießen Ev. Krankenhaus Mittelhessen
Gießen Uni-Kinderklinik
Graz Uni Innere
Graz Uni-Kinderklinik
Greifswald Uni-Kinderklinik
Gummersbach Oberbergklinikum
Görlitz Städtische Kinderklinik
Göttingen Uni Gastroenterologie
Göttingen Uni-Kinderklinik
Hagen Kinderklinik
Halle Uni-Kinderklinik
Hamburg Altonaer Kinderklinik

Hamburg Kinderklinik Wilhelmstift
Hamburg-Nord Kinder-MVZ
Hameln Kinderklinik
Hamm Kinderklinik
Hanau Kinderklinik
Hanau diabetol. Schwerpunktpraxis
Hannover DM-SPP
Hannover Kinderklinik auf der Bult
Haren Kinderarztpraxis
Heide Kinderklinik
Heide Westküstenklinikum Innere Medizin
Heidelberg St. Josefskrankenhaus
Heidelberg Uni-Kinderklinik
Heidenheim Kinderklinik
Heilbronn Innere Klinik
Heilbronn Kinderklinik
Herford Kinderarztpraxis
Herford Klinikum Kinder & Jugendliche
Heringsdorf Inselklinik
Herne Evan. Krankenhaus Innere
Hildesheim Bernward Krks Kinderheilkunde
Hildesheim Kinderarztpraxis
Hof Kinderklinik
Hohenmölsen Diabeteszentrum
Homburg Uni-Kinderklinik Saarland
Idar Oberstein Schwerpunktpraxis

Ingolstadt Klinikum Innere
Innsbruck Uni-Kinderklinik
Innsbruck Universitätsklinik Innere
Itzehoe Kinderklinik
Jena Kinderarztpraxis
Jena Uni-Kinderklinik
Kaiserslautern Kinderarztpraxis
Kaiserslautern-Westpfalzklinikum Kinderklinik
Kamen Klinikum Westfalen Hellmig Krankenhaus
Kamen MKK - Medizinisches Kompetenzkollegium
Karlsburg Klinik für Diabetes & Stoffwechsel
Karlsruhe Schwerpunktpraxis
Karlsruhe Städtische Kinderklinik
Kassel Klinikum Kinder- und Jugendmedizin
Kaufbeuren MVZ für Kinder- und Jugendmedizin
Kempten Oberallgäu Kinderklinik
Kiel Städtische Kinderklinik
Kiel Universitäts-Kinderklinik
Kirchen DRK Krankenhaus Kinderklinik
Kirchheim-Nürtingen Innere
Klagenfurt Kinderklinik
Klagenfurt Klinikum am Wörthersee Abteilung IMuGast
Kleve Innere Medizin
Koblenz Kemperhof 1. Mediz. Klinik
Koblenz Kinderklinik Kemperhof
Konstanz Innere Klinik

Konstanz Kinderklinik
Krefeld Innere Klinik
Krefeld Kinderklinik
Kreischa-Zscheckwitz Klinik Bavaria
Köln Kinderklinik Amsterdamerstrasse
Köln Uni-Kinderklinik
Landau Innere
Landshut Kinderklink
Lappersdorf Kinderarztpraxis
Leer Klinikum - Klinik Kinder & Jugendmedizin
Leipzig Uni-Kinderklinik
Leoben LKH Kinderklinik
Leverkusen Kinderklinik
Lienz Diabetesschwerpunktpraxis für Kinder und Jugendliche
Limburg Innere Medizin
Lindlar DM-Zentrum
Linz KUK MedCampus IV Kinderklinik
Linz Krankenhaus der Barmherzigen Schwestern Kinderklinik
Lippstadt Evangelische Kinderklinik
Ludwigsburg Kinderklinik
Ludwigshafen Kinderklinik St.Anna-Stift
Ludwigshafen diabetol. SPP
Luxembourg - Centre Hospitalier
Lübeck Uni-Kinderklinik
Lübeck Uni-Klinik Innere Medizin
Lüdenscheid Märkische Kliniken - Kinder & Jugendmedizin

Lünen Klinik am Park
Magdeburg Ki-Klinik St. Marienstift
Magdeburg Städtisches Klinikum Innere
Magdeburg Uni-Kinderklinik
Mainz Uni-Kinderklinik
Manderscheid Rathauspraxis
Mannheim Uni-Kinderklinik
Marburg Uni-Kinderklinik
Marktredwitz Innere Medizin
Mechernich Kinderklinik
Meissen Kinderklinik Elblandklinikum
Memmingen Internistische Praxis
Memmingen Kinderklinik
Minden Kinderklinik
Moers Kinderklinik
Mutterstadt Kinderarztpraxis
Mödling Kinderklinik
Mönchengladbach Kinderklinik Rheydt Elisabethkrankenhaus
Mühlheim an der Ruhr Evang. Krankenhaus Med. Klin.
München 3. Orden Kinderklinik
München Kinderarztpraxis diabet. SPP
München Praxiszentrum Saarstrasse
München von Haunersche Kinderklinik
München-Gauting Kinderarztzentrum
München-Schwabing Kinderklinik
Münster Herz Jesu Innere

Münster Ludgerus-Kliniken GmbH
Münster St. Franziskus Innere Med.
Münster St. Franziskus Kinderklinik
Münster Uni-Kinderklinik
Neuburg Kinderklinik
Neumünster Friedrich-Ebert-Kankenhaus Pädiatrie
Neunkirchen Gemeinschaftspraxis Kinderheilkunde
Neunkirchen Innere Medizin
Neunkirchen Marienhausklinik Kohlhof Kinderklinik
Neuss Lukas-Krankenhaus Kinderklinik
Neuwied Kinderklinik Elisabeth
Nidda Bad Salzhausen Klinik Rabenstein/Innere-1 Reha
Nürnberg Cnopfsche Kinderklinik
Nürnberg Uniklinik Med. Klinik 4
Nürnberg Uniklinik Zentrum f Neugeb./Kinder & Jugendl.
Oberhausen Innere
Oberhausen Kinderklinik
Oberhausen Kinderpraxis
Oberhausen St.Clemens Hospitale Sterkrade
Oberwart - Burgenländische Krankenanstalten Pädiatrie
Offenburg Kinderklinik
Oldenburg Kinderklinik
Oldenburg Schwerpunktpraxis Pädiatrie
Olpe pädiatrische Gemeinschaftspraxis
Osnabrück Christliches Kinderhospital
Paderborn St. Vincenz Kinderklinik

Passau Kinderklinik
Pforzheim Kinderklinik
Pirmasens Städtisches Krankenhaus Innere
Plauen Vogtlandklinikum
Prenzlau Krankenhaus Innere
Ravensburg Kinderklink St. Nikolaus
Regensburg Kinderklinik St. Hedwig
Remscheid Kinderklinik
Rendsburg Kinderklinik
Reutlingen Kinderarztpraxis
Reutlingen Kinderklinik
Reutte Tirol BKH Kinderklinik
Rheine Mathiasspital Kinderklinik
Ried Innkreis Barmherzige Schwestern
Rosenheim Innere Medizin
Rosenheim Kinderklinik
Rosenheim Schwerpunktpraxis
Rostock Uni-Kinderklinik
Rotenburg/Wümme Agaplesion Diakonieklinikum Kinderabteilung
Rottweil Gemeinschaftspraxis für Innere Medizin
Ruit-Ostfildern Medius-Klinik Innere
Saaldorf-Surheim Diabetespraxis
Saarbrücken Kinderklinik Winterberg
Salzburg Universitäts-Kinderklinik
Scheibbs Landesklinikum
Scheidegg Prinzregent Luitpold

Schleswig Heliosklinik Kinderklinik
Schw. Gmünd Stauferklinik Kinderklinik
Schweinfurt Kinderklinik
Schwerin Innere Medizin
Schwerin Kinderklinik
Siegen Kinderklinik
Singen Hegau Bodensee-Klinikum Kinderklinik
Singen Kinderarztpraxis
Spaichingen Innere
Speyer Diakonissen Stiftungskrankenhaus Pädiatrie
St. Augustin Kinderklinik
St. Gallen Ostschweizer Kinderspital
St. Johann Tirol Kinderklinik
St. Pölten Universitäts-Kinderklinik
Stade Kinderklinik
Steyr (Pyhrn-Eisenwurzen Klinikum), Abt. Kinder- und Jugendheilkunde
Stockerau Landeskrankenhaus
Stolberg Kinderklinik
Stuttgart Olgahospital Kinderklinik
Suhl Kinderklinik
Sylt Rehaklinik
Tettnang Innere Medizin
Traunstein Kinderklinik
Trier Kinderklinik der Borromäerinnen
Trostberg Innere
Tübingen Uni-Kinderklinik

Ulm Endokrinologikum Amedes
Ulm Uni Innere Medizin
Ulm Uni-Kinderklinik
Vechta Kinderklinik
Viersen Kinderkrankenhaus St. Nikolaus
Villach Kinderklinik
Villingen-Schwenningen Schwarzwald Baar Klinikum Kinderklinik
Villingen-Schwenningen Schwarzwald-Baar-Klinikum Innere
Volkertshausen Gemeinschaftspraxis
Vöcklabruck Kinderklinik
Waldshut Kinderpraxis
Waren-Müritz Kinderklinik
Weiden Kinderklinik
Weingarten Kinderarztpraxis
Weisswasser Kreiskrankenhaus
Wels Klinikum Pädiatrie
Wendelstein Kinder- + Jugendarztpraxis
Wesel Marienhospital Kinderklinik
Wien 3. Med. Hietzing Innere
Wien KH Nord-Klinik Floridsdorf
Wien Klinik Ottakring (Wilhelminenspital) 5. Med. Abteilung
Wien Preyersches Kinderspital
Wien Rudolfstiftung 1. Med. Abtl.
Wien SMZ Ost Donauspital
Wien Uni Innere Med III
Wien Uni-Kinderklinik

Wiener Neustadt Landesklinikum Pädiatrie
Wiesbaden Helios Horst-Schmidt-Kinderkliniken
Wilhelmshaven Kinderarztpraxis
Wilhelmshaven Klinikum Kinderklinik
Winnenden Rems-Murr Kinderklinik
Witten Kinderarztpraxis
Wittenberg Innere Medizin
Wittenberg Kinderklinik
Wittlich DSP
Worms - Weierhof
Worms Kinderklinik
Worms Schwerpunktpraxis
Wuppertal Universitäts-Kinderklinik
Würzburg Uni-Kinderklinik
Zams Kinderklinik
Zweibrücken Kinderarztpraxis
Zwettl Landesklinikum Gmünd-Waidhofen
Zwettl Landesklinikum Gmünd-Waidhofen Kinderklinik
