# Supplementary material for: Highly Uncontrolled Cardiovascular Risk in Emerging Adults With Paediatric‐Onset Type 1 Diabetes—A Cross‐Sectional Analysis From the Diabetes Prospective Follow‐Up Registry DPV
Source: Diabetes Obes Metab. 2026 Mar 16;28(5):4194–204. doi: 10.1111/dom.70610 (PMC13071203; doi:10.1111/dom.70610)
Supplement: Supplementary file 2 — Table S1: Comparison of included individuals with complete documentation of all five predefined CV risk factors and excluded cases with incomplete risk factor documentation. Data are presented as medians with interquartile ranges (Q1–Q3) or percentages. Group differences were assessed using Wilcoxon tests for continuous variables and chi‐square tests for categorical variables; corresponding p‐values are shown. [file DOM-28-4194-s002.pdf]

**Supplementary Table 1:**	**patients with all 5 risk factors documented**		**excluded patients (incomplete risk factor documentation)**		
	**n**	**median (Q1-Q3) or %**	**n**	**median (Q1-Q3) or %**	**p**
age (years)	7,298	17.8 (17.5-18.5)	3,745	17.8 (17.4-20.2)	0.011
male	7,298	53.9	3,745	54.4	n.s.
Age at diabetes onset (years)	7,298	9.6 (6.0-12.6)	3,745	9.8 (6.0-12.8)	n.s.
diabetes duration (years)	7,298	8.5 (5.5-12.2)	3,745	9.2 (5.8-13.1)	<0.001
migrational background	7,298	23.5	3,745	21.7	n.s.
BMI (kg/m2)	7,298	23.7 (21.5-26.5)	3,657	23.6 (21.4-26.6)	n.s.
overweight (BMI >25 kg/m2)	7,298	25.3	3,745	21.5	<0.001
**obesity (BMI >30 kg/m2)**	**7,298**	**7.5**	**3,745**	**7.0**	**n.s.**
systolic blood pressure (mmHg)	7,298	126 (120-132)	3,745	126 (119-134)	n.s.
diastolic blood pressure (mmHg)	7,298	75 (70-80)	3,745	76 (70-82)	<0.001
**elevated blood pressure (>130/80 mmHG)**	**7,298**	**17.6**	**3,745**	**22.9**	**<0.001**
arterial hypertension (>140/90 mmHg)	7,298	1.8	3,745	4.1	<0.001
total cholesterol (mg/dL)	7,224	168 (147-191)	826	172 (151-196)	0.003
LDL cholesterol (mg/dL)	7,298	96 (78-116)	561	103 (82-126)	<0.001
HDL cholesterol (mg/dL)	7,214	57 (49-67)	636	57 (47-68)	n.s.
non-HDL cholesterol (mg/dL)	7,151	109 (89-131)	631	116 (93-142)	<0.001
triglycerides (mg/dL)	7,110	92 (66-134)	793	93 (66-139)	n.s.
LDL cholesterol >100 mg/dL	7,298	44.3	5,61	52.9	0.001
**LDL cholesterol >130 mg/dL**	**7,298**	**14.1**	**5,61**	**21.9**	**<0.001**
HbA1c (%)	7,298	7.9 (7.2-8.9)	3,623	8.0 (7.2-9.2)	<0.001
HbA1c >7 % (>53 mmol/mol)	7,298	79.8	3,623	79.5	n.s.
**HbA1c >9% (>75 mmol/mol)**	**7,298**	**21.8**	**3,623**	**27.3**	**<0.001**
**smoker**	**7,298**	**13.3**	**3,745**	**14.6**	**n.s.**
diabetic retinopathy	4,306	0.3	1,309	0.76	n.s.
microalbuminuria	6,012	10	1,404	13.4	0.005
